# Traumatic birth experiences and maternal caregiving behaviors and attitudes in black and white women

**DOI:** 10.1007/s00737-026-01682-6

**Published:** 2026-03-19

**Authors:** Sara L. Kornfield, Nicole M. Henry, Rebecca Waller, Lauren White, Raquel Gur, Deiriai Myers, Kate Wisniewski, Florence Momplaisir, Wanjikũ F. M. Njoroge

**Affiliations:** 1 Department of Psychiatry, Center for Women’s Behavioral Wellness, University of Pennsylvania, Philadelphia, PA USA; 2 Lifespan Brain Institute (LiBI), Children’s Hospital of Philadelphia and Penn Medicine, Philadelphia, PA USA; 3 Department of Child and Adolescent Psychiatry and Behavioral Sciences, Children’s Hospital of Philadelphia, Philadelphia, PA USA; 4 PolicyLab, Children’s Hospital of Philadelphia, Philadelphia, PA USA; 5 Psychology Department, University of Pennsylvania, Philadelphia, PA USA; 6 Department of Child and Adolescent Psychiatry and Behavioral Sciences, Children’s Hospital of Philadelphia, Philadelphia, PA USA; 7 Department of Psychiatry, University of Pennsylvania, Philadelphia, PA USA; 8 Department of Medicine, Division of Infectious Diseases, University of Pennsylvania, Philadelphia, PA USA; 9 Penn Leonard Davis Institute of Health Economics, University of Pennsylvania, Philadelphia, PA USA

**Keywords:** Childbirth trauma, Caregiving, Women, Coronavirus (COVID-19) pandemic

## Abstract

**Purpose:**

This longitudinal investigation examined the association between traumatic birth experiences (measured via self-report and clinician-report) and caregiving behaviors and attitudes and any race-related differences in these associations.

**Methods:**

Subjective childbirth trauma was measured via a three-item questionnaire at 12 weeks postpartum. Medical childbirth factors were extracted from the electronic health record. Maternal caregiving behaviors and attitudes were assessed via comprehensive questionnaires (i.e., mother-infant bonding and parenting stress) and observation ratings (i.e., positive parenting and mother-infant interactions) at 12 weeks, 12 months, and 24 months postpartum. Multiple linear regressions were run to analyze these relationships.

**Results:**

A total of 255 mothers (106 Black and 149 White) who gave birth from April to December 2020 were examined. More traumatic childbirth experiences were significantly associated with higher-rated observed positive parenting scores (*β* = 0.21, *p*_*FDR*_<0.05) when controlling for demographic factors. There were no significant relationships at 12 weeks or 24 months postpartum. Additionally, there were no effects of race on the relationship between childbirth trauma and caregiving.

**Conclusions:**

Subjective reports of childbirth trauma were not significantly associated with poorer maternal caregiving behaviors and attitudes. This study adds to the literature by examining Black women, as they are underrepresented in this body of research and more at risk of experiencing traumatic childbirths.

**Supplementary Information:**

The online version contains supplementary material available at 10.1007/s00737-026-01682-6.

## Highlights

This study investigated the relationship between childbirth trauma and various maternal caregiving behaviors, measured at several timepoints during the postpartum period. The sample included Black and White women who gave birth early in the COVID-19 pandemic, a time of heightened stress for those delivering in a hospital setting.

We found mostly nonsignificant relationships but one positive relationship between childbirth and caregiving behaviors when children were 12 months old: More traumatic birth experiences were related to better caregiving scores.

This is the first study to focus on Black individuals’ childbirth experiences and its relation to caregiving behaviors. More research is needed on women of color, as they have a higher risk of experiencing a traumatic birth.

**Supplementary Information:**

The online version contains supplementary material available at 10.1007/s00737-026-01682-6.

## Introduction

The experience of childbirth as traumatic is common and has a high likelihood of impacting postpartum maternal well-being and the long term maternal-child relationship (Lewkowitz et al. 2024; Vega-Sanz et al. 2025). During the COVID-19 pandemic, reports of childbirth trauma and birth-related PTSD symptoms increased significantly, with 33.7% reported prevalence of postpartum PTSD (Gao et al. 2022), compared to 2.9–18.5% pre-pandemic rates in community and at-risk samples, respectively (Grekin and O’Hara 2014; Heyne et al. 2022; Yildiz et al. 2017). The most recent data from the Maternal Mental Health Leadership Alliance suggests that 1 in 3 birthing people report traumatic birth experiences (Ayers and Pickering 2001; Maternal Mental Health Leadership Alliance, 2025; Pidd et al. 2023).

Childbirth can be objectively traumatic, such as in situations where a mother’s and/or the baby’s life is at risk (e.g., emergency cesarean section, postpartum hemorrhage; Horsch et al. 2024). Nonetheless, childbirth experiences are highly subjective (i.e., the degree to which a woman feels like her life or child’s life is in danger; Beck 2004; Horsch et al. 2024); even non-life-threatening situations including negative attitudes and treatment by healthcare workers, miscommunication, emotional neglect, difficulty advocating, unexplained procedures, and (perceived or real) rushed decision-making can contribute to a childbirth experience being perceived as traumatic (Mergler et al. 2025). Similarly, medical complexities noted in the health record, including postpartum hemorrhage, caesarean delivery, and preterm birth, contribute directly and indirectly to perceptions of trauma during childbirth and subsequent postpartum depression. Thus, self-reported trauma is a significant predictor of postpartum maternal functioning even in medically “routine” birth (Waller et al. 2022).

Beyond maternal mental health outcomes, traumatic birth experiences and birth-related PTSD symptoms have been linked to subsequent maternal caregiving difficulties (Bell et al. 2018a, b; Christie et al. 2019), which is concerning as positive caregiving behaviors are critical to children’s development and well-being (Frosch et al. 2019). Indeed, birth-related PTSD has been associated with disruptions to mother-infant bonding and attachment (Ballard et al. 1995; Frankham et al. 2024; Sigurðardóttir et al. 2025). However, findings have generally been equivocal with some reporting no association (Handelzalts et al. 2021; Stuijfzand et al. 2020). Inconsistent findings may be due to short term follow-up, generally within 6 months of birth, resulting in limited knowledge of the long-term implications of maternal childbirth trauma on maternal-infant bonding and behaviors. Furthermore, reliance on self-report measures limits findings to what parents choose to report regarding their own attitudes about parenting and bonding and are susceptible to social desirability bias (Zahidi et al. 2019).

Studies in this area have often failed to include women of color, even as Black women are more likely to report childbirth trauma than White women (Iyengar et al. 2022; Waller et al. 2022). Moreover, research on birth trauma and subsequent caregiving has primarily examined samples of highly educated White women (Bell et al. 2018a, b), making the findings difficult to generalize to those most likely to report trauma. Minoritized women have consistently reported significantly lower birth satisfaction than White women prior to (Breman et al. 2021; Hamm et al. 2020; Janevic et al. 2021) and during the pandemic (Mollard and Kupzyk 2022).

To investigate the relationship between birth trauma and maternal caregiving behaviors and attitudes, considering the differential impact of race, we examined a cohort of Black and White women who gave birth from April to December 2020. To address gaps in the current literature, we used longer term follow-up periods (up to 24 months) and leveraged self-report and observed measures of parenting behaviors and attitudes. In addition to self-reported perceptions of traumatic childbirth, we also included clinician-reported data about childbirth factors extracted from the patients’ medical charts.

We hypothesized that those who self-report more traumatic birth experiences, measured at 12 weeks postpartum, would report less positive maternal caregiving behaviors and attitudes at 12 and 24 months postpartum. Similarly, we expected that those with more clinician-reported childbirth factors (i.e., blood loss, caesarean birth, preterm birth, etc.) would also display less positive maternal caregiving behaviors and attitudes. Lastly, we hypothesized that there would be differences in the relationship between birth trauma and maternal caregiving and attitudes by race.

## Materials and methods

### Design and procedures

This study reports on a longitudinal investigation conducted from 2020 to 2023. Pregnant women were recruited by targeted emails during pregnancy and gave birth during the early pandemic (i.e., April to December 2020) at an urban East coast hospital system. At 12 weeks postpartum (*M* = 12.3, *SD* = 4.37) (T1), participants completed demographic, subjective birth experience, caregiving, and mental health questionnaires. Around 12 months (*M* = 13.7, *SD* = 1.12) (T2), mothers participated in interactive online visits where they and their baby completed several tasks in their homes, administered by research assistants via video-conferencing technology. Visits were recorded and double-coded by trained research staff to assess the quality of maternal caregiving. Participants also completed surveys at 12 and 24 months (*M* = 26.7, *SD* = 0.96) (T3) about their mental health and additional demographic information. Electronic health records (EHR) were obtained for labor and delivery information. A total of 255 participants were included in the analyses. One-hundred-six (41.6%) mothers were Black and 149 (58.4%) were White. The mean maternal age at birth was 33.0 years (*SD* = 4.97). There were several significant baseline differences. See Table 1 for demographic, self-report survey, and delivery information.

**Table 1:** Group difference of sociodemographic information

Variable	Black (*N* = 106)	White (*N* = 149)	*p*
Maternal age at delivery			< 0.001***
Mean (SD)	31.0 (5.63)	34.4 (3.86)	
Median [Min, Max]	30.3 [18.8, 46.3]	34.2 [24.7, 45.7]	
Income level (T1)			< 0.001***
≥ $100K	12 (11.3%)	118 (79.2%)	
< $100K	94 (88.7%)	31 (20.8%)	
Education (T1)			< 0.001***
High school degree or lower	31 (29.2%)	4 (2.7%)	
Some college or above	75 (70.8%)	145 (97.3%)	
Relationship status (T1)			< 0.001***
Married/cohabiting	58 (54.7%)	144 (96.6%)	
Single/Widowed/Separated	46 (43.4%)	5 (3.4%)	
Missing	2 (1.9%)	0 (0%)	
Child sex			0.334
Female	46 (43.4%)	75 (50.3%)	
Male	60 (56.6%)	74 (49.7%)	
Clinician-reported adverse childbirth events			0.239
Mean (SD)	0.802 (0.844)	0.685 (0.736)	
Median [Min, Max]	1.00 [0, 4.00]	1.00 [0, 3.00]	
Preterm birth			0.626
Gestational age **≥** 37 weeks	100 (94.3%)	137 (91.9%)	
Gestational age < 37 weeks	6 (5.7%)	12 (8.1%)	
Delivery method			0.604
Vaginal	78 (73.6%)	104 (69.8%)	
Caesarean	28 (26.4%)	45 (30.2%)	
Placental removal method			1.000
Expressed or Spontaneous	101 (95.3%)	141 (94.6%)	
Manual	5 (4.7%)	8 (5.4%)	
5 min Apgar score			0.008**
5-min Apgar Score > 8	91 (85.8%)	142 (95.3%)	
5-min Apgar Score ≤ 8	15 (14.2%)	6 (4.0%)	
Missing	0 (0%)	1 (0.7%)	
Blood loss (mL)			0.691
Mean (SD)	330 (173)	340 (158)	
Median [Min, Max]	300 [100, 1200]	300 [100, 1100]	
Missing	34 (32.1%)	52 (34.9%)	
Labor analgesia			0.131
Received labor analgesia (e.g., general, spinal, epidural)	92 (86.8%)	138 (92.6%)	
No labor analgesia	13 (12.3%)	9 (6.0%)	
Missing	1 (0.9%)	2 (1.3%)	
Assisted vaginal births and/or episiotomy			0.239
Spontaneous vaginal or caesarean birth	70 (66.0%)	87 (58.4%)	
Assisted vaginal births and/or episiotomy	8 (7.5%)	4 (2.7%)	
Missing	28 (26.4%)	58 (38.9%)	
Lacerations/tearing			0.677
No lacerations/tearing or 1st to 2nd degree	83 (78.3%)	106 (71.1%)	
3rd degree or more	2 (1.9%)	5 (3.4%)	
Missing	21 (19.8%)	38 (25.5%)	
Self-reported childbirth trauma			< 0.001***
Mean (SD)	6.12 (2.70)	4.87 (2.03)	
Median [Min, Max]	6.00 [3.00, 10.9]	4.00 [3.00, 10.9]	
Mother-infant bonding (T1)			0.001**
Mean (SD)	4.20 (3.49)	5.86 (4.24)	
Median [Min, Max]	4.00 [0, 13.9]	5.00 [0, 13.9]	
Missing	2 (1.9%)	0 (0%)	
Observed mother-infant interactions (T2)			0.252
Mean (SD)	0.0817 (0.753)	-0.0556 (0.756)	
Median [Min, Max]	0.120 [-1.76, 1.39]	-0.0860 [-1.76, 2.03]	
Missing	36 (34.0%)	56 (37.6%)	
Observed positive parenting (T2)			0.016*
Mean (SD)	-0.130 (1.05)	0.201 (0.743)	
Median [Min, Max]	-0.117 [-2.18, 1.80]	0.162 [-1.50, 2.17]	
Missing	31 (29.2%)	52 (34.9%)	
Parenting stress (T3)			0.926
Mean (SD)	102 (18.6)	101 (17.1)	
Median [Min, Max]	98.0 [70.0, 138]	100 [71.0, 138]	
Missing	23 (21.7%)	21 (14.1%)	
Postpartum internalizing symptoms (T1)			0.997
Mean (SD)	-0.000272 (1.14)	0.000194 (0.796)	
Median [Min, Max]	-0.341 [-1.17, 3.40]	-0.186 [-1.17, 2.59]	

* indicates *p* < 0.05, ** indicates *p* < 0.01, *** indicates *p* < 0.001. T1 = 12 weeks postpartum, T2 = 12 months postpartum, and T3 = 24 months postpartum. Mother infant bonding was assessed via the general impairment subscale of the Postpartum Bonding Questionnaire (PBQ), and parenting stress was measured via the Parenting Stress Index, fourth edition, short form (PSI-4-SF). Birth trauma was assessed via questionnaire created by the study’s authors. Postpartum internalizing symptoms were assessed via a composite score of the Edinburgh Postnatal Depression Scale (EPDS) and Generalized Anxiety Disorder 7 (GAD-7). Mother-infant interactions and positive parenting were assessed via observational reports. The cumulative score of clinician-reported adverse childbirth events was derived based on the number of objective traumatic birth events recorded in the medical chart (i.e., caesarean-section, preterm-birth, significant blood loss, manual placental removal method, an Apgar score of less than 8, no labor analgesia, assisted vaginal births and/or episiotomy, *≥*3rd degree lacerations/tearing)

The Institutional Review Boards at the Children's Hospital of Philadelphia and the University of Pennsylvania approved this study. See Njoroge et al (2024) and Waller et al (2022) for additional study information.

### Measured variables

See Table 1 and Table S1 for descriptive statistics and additional details of all study variables.

#### Race

At T2 and T3, mothers reported their racial identity. A total of 44 participants did not report their race in the T2 and/or T3 survey. For these participants, race was identified from EHR. One caveat with using EHR for racial identification is that there is not always a standardized method of obtaining this information (Kim et al. 2022).

#### Self-reported birth trauma

Self-reported birth trauma was reported from the sum of three questions on a scale of 1 (*Not at all*) to 4 (*Extremely/a lot*): “During your delivery or immediately after delivery,” (1) “How much did you fear for your own life?,” (2) “How much did you fear for your baby’s life?,” and (3) “How traumatic did you find your birth experience?” These questions were based on Criterion A from the DSM-5 diagnostic criteria for PTSD (American Psychiatric Association, 2013) which specifies exposure to threatened death to self or a loved one as required criteria for the diagnosis of posttraumatic stress disorder. Items were developed via multiple iterative consultations with clinical experts on our study team to finalize a brief set of survey questions that captures self-reported traumatic childbirth experiences (e.g., Abdollahpour et al. 2017; Hyland et al. 2018; Waller et al. 2022). Higher scores indicate more traumatic birth experiences. Internal validity was good (Cronbach’s alpha=0.76), and the measure was significantly correlated to clinician-reported adverse birth events (See Table S2). See Waller et al. (2022) for additional details.

#### Adverse clinician-reported birth events

A cumulative score of clinician-reported adverse childbirth events was derived based on the number of objective traumatic birth events recorded in the medical chart, including caesarean-section, no labor analgesia, preterm-birth, significant levels of blood loss (> 400mL for vaginal births, > 1000mL for caesarean-section births), manual placental removal method (for vaginal births only), a 5-minute Apgar score of less than 8, *≥*3rd degree lacerations/tearing, and assisted vaginal births and/or episiotomy. Inclusion of these events was based on earlier analysis using the same sample (Waller et al. 2022) and was guided by the input of experts in obstetrics/neonatology. Each adverse birth event was coded as 0 (i.e., the mother did not experience an adverse birth event) or 1 (i.e., the mother did experience an adverse birth event). These values were added together to create a cumulative score of clinician-reported adverse childbirth events, where higher scores indicates that a participant experienced more clinician-reported adverse childbirth events.

#### Self-reported mother-infant bonding

The “general impairment” subscale of the Postpartum Bonding Questionnaire (PBQ) and was assessed at T1 (Brockington et al. 2006). The subscale consists of 12 questions that can be scored from 1 (*Always*) to 6 (*Never*) and was assessed at T1. Higher scores indicate greater mother-infant bonding problems. Internal validity was good (Cronbach’s alpha=0.80).

#### Observed mother-infant interactions

Coders assessed mother-infant interactions from video recordings of mothers and their children engaging in two tasks (i.e., storybook, free play). Interactions were assessed at T2, and this construct measures the level of synchrony, comfort, and mutual pleasure between parent and child while participating in the tasks. Coders rated interactions on a scale 0 (*Very low*) to 7 (*Very high*) for both tasks (ICC=0.77-0.79). Scores were combined to create a latent score of observed mother-infant interaction. Low scores indicate that a dyad operates as two separate entities, while high scores indicate a high degree of synchrony and comfort.

#### Observed positive parenting

Positive parenting was measured at T2 via the video recordings and was assessed using ratings from the three tasks. The three facets of positive parenting include maternal sensitivity, positive regard, and stimulation of cognitive development. Coders rated each facet on a scale of 0 (*Very low*) to 7 (*Very high*) for all three tasks (ICC=0.72-0.87). A confirmatory factor analysis derived a latent score of observed positive parenting. Higher scores indicate high levels of positive parenting. See Waller et al. (2024) for additional psychometric details.

#### Parenting stress

The Parenting Stress Index, fourth edition, short form (PSI-4-SF) measures the amount of stress present in parent-child relationships. This short form uses 36 items from the original version of the PSI (Barroso et al. 2016). Participants rated items on a scale of 1 (*Strongly agree)* to 5 (*Strongly disagree*). Higher scores indicate more parenting stress. Internal validity was excellent (Cronbach’s alpha=0.92).

#### Postpartum internalizing symptoms

Postpartum internalizing symptoms were measured via a continuous composite score of depression and anxiety symptoms. Depression was measured via the Edinburgh’s Postpartum Depression Scale (EPDS; Cox 2017; Cox et al. 1987) (Cronbach’s alpha=0.87). Anxiety was measured via the Generalized Anxiety Disorder 7-item scale (GAD-7; Spitzer et al. 2006) (Cronbach’s alpha=0.91). Because the EPDS and GAD-7 were highly correlated (*r*=0.83, *p*<0.001), the questionnaires were standardized and averaged together to create a composite postpartum internalizing symptoms score.

#### Sociodemographic information

The following variables were used to characterize the current sample and/or examined as covariates: baseline reported income and education, marital status, age at delivery, and child’s age at each assessment timepoint.

### Analytic strategy

All analyses were conducted in R version 4.5.1. We performed a series of linear regression models examining the relationship of maternal caregiving behaviors and attitudes with self-reported childbirth trauma (Aim 1) and clinician-reported adverse childbirth events (Aim 2). The outcomes for the regression models were mother-infant bonding (T1), observed mother-infant interactions (T2), observed positive parenting (T2, T3) and parenting stress (T3). A follow-up, exploratory analysis examined the interaction between postpartum internalizing symptoms and subjective birth trauma and its impact on caregiving behaviors. To do this, the interaction term was added to all linear regression models described above. Lastly, we examined the interactive effect between childbirth trauma (both self-reported and clinician-reported) and race on maternal caregiving behaviors and attitudes. Given the number of tests performed, the False Discovery Rate (FDR) procedure was used to adjust for multiple comparisons. Participants with incomplete or full data were included in analyses. In the case of missing values, a participant’s mean ratings of items were used if no more than 20% of the questions were missing. Listwise deletion per model was used to handle missing data. Self-reported measures were winsorized to address outliers, and observation measures were standardized.

## Results

### Aim 1a: The relationship between self-reported birth trauma and maternal caregiving

See Table S2 for correlations of all study variables. A significant association between self-reported birth trauma and positive parenting (T2) emerged when adjusting for covariates and held when controlling for multiple corrections (*β* = 0.21, *p*<0.01, *p*_*FDR*_<0.05), where greater childbirth trauma was associated with higher observed positive parenting scores. No other significant relationships were detected. See Table 2.

**Table 2:** Regressions examining the relationship between self-reported childbirth trauma and subsequent caregiving behaviors

Dependent Variable	Predictor	Model 1					Model 2				
		*B*	*SE*	*β*	*p*	*FDR p*	*B*	*SE*	*β*	*p*	*FDR p*
Mother-infant bonding (T1)	Race (0 = White, 1 = Black)	-1.61	0.60	-0.20	0.008**	0.031*	-1.46	1.34	-0.20	0.276	0.459
	Relationship status	0.64	0.72	0.06	0.371	0.371	0.65	0.72	0.07	0.368	0.459
	Child age (months)	-0.24	0.25	-0.06	0.332	0.371	-0.24	0.25	-0.06	0.335	0.459
	Self-reported childbirth trauma	0.17	0.11	0.10	0.123	0.246	0.18	0.16	0.10	0.254	0.459
	Self-reported childbirth trauma × Race	-	-	-	-	-	-0.03	0.22	-0.01	0.899	0.899
Observed mother-infant interactions (T2)	Race (0 = White, 1 = Black)	0.17	0.14	0.11	0.235	0.314	-0.11	0.33	0.11	0.740	0.805
	Relationship status	0.22	0.16	0.12	0.175	0.314	0.21	0.16	0.11	0.204	0.805
	Child age (months)	-0.01	0.06	-0.02	0.811	0.811	-0.01	0.06	-0.02	0.805	0.805
	Self-reported childbirth trauma	0.05	0.03	0.14	0.092	0.314	0.02	0.04	0.12	0.703	0.805
	Self-reported childbirth trauma × Race	-	-	-	-	-	0.05	0.05	0.08	0.349	0.805
Positive parenting (T2)	Race (0 = White, 1 = Black)	-0.38	0.16	-0.21	0.018*	0.037*	-0.64	0.37	-0.21	0.087	0.435
	Relationship status	0.16	0.18	0.07	0.374	0.499	0.15	0.18	0.07	0.418	0.551
	Child age (months)	0.03	0.06	0.04	0.579	0.579	0.03	0.06	0.04	0.575	0.575
	Self-reported childbirth trauma	0.08	0.03	0.21	0.009**	0.036*	0.05	0.05	0.19	0.278	0.551
	Self-reported childbirth trauma × Race	-	-	-	-	-	0.05	0.06	0.06	0.441	0.551
Parenting stress (T3)	Race (0 = White, 1 = Black)	1.57	2.89	0.04	0.588	0.601	3.17	6.79	0.05	0.641	0.847
	Relationship status	2.60	3.69	0.06	0.482	0.601	2.76	3.75	0.06	0.463	0.847
	Child age (months)	-1.13	1.29	-0.06	0.380	0.601	-1.13	1.29	-0.06	0.382	0.847
	Self-reported childbirth trauma	-0.28	0.54	-0.04	0.601	0.601	-0.15	0.75	-0.04	0.847	0.847
	Self-reported childbirth trauma × Race	-	-	-	-	-	-0.28	1.09	-0.02	0.795	0.847

* indicates *p* < 0.05, ** indicates *p* < 0.01, *** indicates *p* < 0.001. *FDR p* = False discovery rate *p*-values. T1 = 12 weeks postpartum, T2 = 12 months postpartum, and T3 = 24 months postpartum. Mother infant bonding was assessed via the general impairment subscale of the Postpartum Bonding Questionnaire (PBQ), and parenting stress was measured via the Parenting Stress Index, fourth edition, short form (PSI-4-SF). Postpartum internalizing symptoms were assessed via a composite score of the Edinburgh Postnatal Depression Scale (EPDS) and Generalized Anxiety Disorder 7 (GAD-7). Mother-infant interactions and positive parenting were assessed via observational reports. Relationship status (1 = married/partnered, 0 = single/divorced/separated/widowed) and race (1 = Black, 0 = White) were dummy coded

In a follow-up analysis we investigated the role of postpartum depression and anxiety, averaged to create a composite variable of postpartum internalizing symptoms, in the relationship between self-reported birth trauma and caregiving behaviors. There was one interaction between birth trauma and postpartum internalizing symptoms when predicting positive parenting scores at T1, but this did not hold when controlling for multiple comparisons. No other significant interactions were observed. See Table 3.

**Table 3:** Regressions examining the relationship between self-reported childbirth trauma, postpartum internalizing symptoms, and subsequent caregiving behaviors

Dependent Variable	Predictor	B	SE	β	*p*	FDR *p*
Mother-infant bonding (T1)	Race (0 = White, 1 = Black)	-1.46	0.57	-0.18	0.011*	0.064
	Relationship status	0.58	0.69	0.06	0.398	0.425
	Child age (months)	-0.41	0.24	-0.10	0.085	0.255
	Internalizing symptoms (T1)	0.66	0.58	0.32	0.260	0.390
	Self-reported birth trauma	0.08	0.10	0.05	0.425	0.425
	Self-reported birth trauma × Internalizing symptoms (T1)	0.13	0.09	0.07	0.168	0.337
Observed mother-infant interactions (T2)	Race (0 = White, 1 = Black)	0.16	0.14	0.10	0.273	0.546
	Relationship status	0.21	0.17	0.11	0.213	0.546
	Child age (months)	-0.02	0.06	-0.02	0.789	0.789
	Internalizing symptoms (T1)	0.05	0.15	-0.03	0.750	0.789
	Self-reported birth trauma	0.05	0.03	0.16	0.076	0.458
	Self-reported birth trauma × Internalizing symptoms (T1)	-0.01	0.02	-0.04	0.576	0.789
Observed positive parenting (T2)	Race (0 = White, 1 = Black)	-0.34	0.16	-0.19	0.034*	0.051
	Relationship status	0.25	0.18	0.11	0.171	0.205
	Child age (months)	0.05	0.06	0.06	0.416	0.416
	Internalizing symptoms (T1)	-0.35	0.16	-0.01	0.034*	0.051
	Self-reported birth trauma	0.07	0.03	0.19	0.023*	0.051
	Self-reported birth trauma × Internalizing symptoms (T1)	0.06	0.02	0.16	0.012*	0.051
Parenting stress (T3)	Race (0 = White, 1 = Black)	1.61	2.74	0.05	0.558	0.836
	Relationship status	3.67	3.57	0.08	0.305	0.609
	Child age (months)	-0.25	1.23	-0.01	0.839	0.959
	Internalizing symptoms (T1)	6.03	2.80	0.33	0.032*	0.194
	Self-reported birth trauma	-0.84	0.52	-0.11	0.112	0.336
	Self-reported birth trauma × Internalizing symptoms (T1)	0.02	0.45	0.00	0.959	0.959

* indicates *p* < 0.05, ** indicates *p* < 0.01, *** indicates *p* < 0.001. *FDR p* = False discovery rate *p*-values. T1 = 12 weeks postpartum, T2 = 12 months postpartum, and T3 = 24 months postpartum. Mother infant bonding was assessed via the general impairment subscale of the Postpartum Bonding Questionnaire (PBQ), and parenting stress was measured via the Parenting Stress Index, fourth edition, short form (PSI-4-SF). Birth trauma was assessed via questionnaire created by the study’s authors. Postpartum internalizing symptoms were assessed via a composite score of the Edinburgh Postnatal Depression Scale (EPDS) and Generalized Anxiety Disorder 7 (GAD-7). Mother-infant interactions and positive parenting were assessed via observational reports. Relationship status (1 = married/partnered, 0 = single/divorced/separated/widowed) and race (1 = Black, 0 = White) were dummy coded

### Aim 1b: Race-related differences in the relationship between self-reported birth trauma on maternal caregiving

As previously reported in Waller et al. 2022, Black women reported significantly greater traumatic childbirth experiences than White participants (*p*<0.001). There were no significant interactions between childbirth trauma and race on maternal caregiving behaviors and attitudes. See Table 2.

### Aim 2a: The relationship between clinician-reported adverse birth events and maternal caregiving

Clinician-reported adverse birth events were not significantly associated with any maternal caregiving outcome. See Table 4.

**Table 4:** Regressions examining the relationship between clinician-reported adverse birth events and subsequent caregiving behaviors

Dependent Variable	Predictor	Model 1					Model 2				
		*B*	*SE*	*β*	*p*	*FDR p*	*B*	*SE*	*β*	*p*	*FDR p*
Mother-infant bonding (T1)	Race (0 = White, 1 = Black)	-1.44	0.59	-0.18	0.015*	0.059	-1.05	0.75	-0.18	0.164	0.406
	Relationship status	0.72	0.72	0.07	0.319	0.425	0.69	0.72	0.07	0.342	0.406
	Child age (months)	-0.20	0.25	-0.05	0.429	0.429	-0.22	0.25	-0.05	0.391	0.406
	Clinician-reported adverse birth events	0.34	0.32	0.07	0.285	0.425	0.59	0.44	0.07	0.178	0.406
	Clinician-reported adverse birth events × Race	-	-	-	-	-	-0.53	0.64	-0.05	0.406	0.406
Observed mother-infant interactions (T2)	Race (0 = White, 1 = Black)	0.24	0.14	0.15	0.091	0.363	0.11	0.18	0.16	0.534	0.667
	Relationship status	0.22	0.16	0.12	0.185	0.370	0.23	0.16	0.12	0.162	0.432
	Child age (months)	-0.01	0.06	-0.02	0.828	0.828	-0.01	0.06	-0.02	0.835	0.835
	Clinician-reported adverse birth events	-0.05	0.08	-0.06	0.477	0.637	-0.15	0.11	-0.08	0.189	0.432
	Clinician-reported adverse birth events × Race	-	-	-	-	-	0.17	0.15	0.09	0.259	0.432
Positive parenting (T2)	Race (0 = White, 1 = Black)	-0.26	0.16	-0.14	0.099	0.395	-0.40	0.20	-0.14	0.050*	0.248
	Relationship status	0.17	0.19	0.08	0.347	0.694	0.18	0.19	0.08	0.319	0.532
	Child age (months)	0.03	0.06	0.04	0.621	0.706	0.03	0.06	0.04	0.616	0.616
	Clinician-reported adverse birth events	0.03	0.09	0.03	0.706	0.706	-0.07	0.13	0.01	0.573	0.616
	Clinician-reported adverse birth events × Race	-	-	-	-	-	0.19	0.18	0.08	0.275	0.532
Parenting stress (T3)	Race (0 = White, 1 = Black)	1.14	2.82	0.03	0.687	0.687	3.58	3.71	0.03	0.337	0.479
	Relationship status	2.50	3.67	0.06	0.497	0.662	2.44	3.67	0.06	0.508	0.508
	Child age (months)	-1.24	1.28	-0.07	0.337	0.662	-1.13	1.29	-0.06	0.383	0.479
	Clinician-reported adverse birth events	1.52	1.58	0.07	0.340	0.662	2.85	2.06	0.07	0.169	0.479
	Clinician-reported adverse birth events × Race	-	-	-	-	-	-3.25	3.23	-0.07	0.314	0.479

* indicates *p* < 0.05, ** indicates *p* < 0.01, *** indicates *p* < 0.001. *FDR p* = False discovery rate *p*-values. T1 = 12 weeks postpartum, T2 = 12 months postpartum, and T3 = 24 months postpartum. Mother infant bonding was assessed via the general impairment subscale of the Postpartum Bonding Questionnaire (PBQ), and parenting stress was measured via the Parenting Stress Index, fourth edition, short form (PSI-4-SF). Postpartum internalizing symptoms were assessed via a composite score of the Edinburgh Postnatal Depression Scale (EPDS) and Generalized Anxiety Disorder 7 (GAD-7). Mother-infant interactions and positive parenting were assessed via observational reports. Relationship status (1 = married/partnered, 0 = single/divorced/separated/widowed) and race (1 = Black, 0 = White) were dummy coded. The cumulative score of clinician-reported adverse childbirth events was derived based on the number of objective traumatic birth events recorded in the medical chart (i.e., caesarean-section, preterm-birth, significant blood loss, manual placental removal method, an Apgar score of less than 8, no labor analgesia, assisted vaginal births and/or episiotomy, and *≥*3rd degree lacerations/tearing)

As above, postpartum internalizing symptoms (T1) were examined as a moderator in the relationship between clinician-reported adverse birth events and maternal caregiving. There was a significant interaction between clinician-reported adverse birth events and postpartum internalizing symptoms; however, this interaction did not survive FDR correction. See Table 5.

**Table 5:** Regressions examining the relationship between clinician-reported adverse birth events, postpartum internalizing symptoms, and subsequent caregiving behaviors

Dependent Variable	Predictor	B	SE	β	*p*	FDR *p*
Mother-infant bonding (T1)	Race (0 = White, 1 = Black)	-1.42	0.56	-0.17	0.011*	0.034*
	Relationship status	0.48	0.68	0.05	0.481	0.577
	Child age (months)	-0.37	0.24	-0.09	0.131	0.263
	Internalizing symptoms (T1)	1.29	0.32	0.33	< 0.001*	0.001*
	Clinician-reported adverse birth events (T1)	0.24	0.30	0.05	0.419	0.577
	Clinician-reported adverse birth events × Internalizing symptoms (T1)	0.14	0.25	0.03	0.579	0.579
Observed mother-infant interactions (T2)	Race (0 = White, 1 = Black)	0.24	0.14	0.15	0.093	0.522
	Relationship status	0.22	0.16	0.12	0.174	0.522
	Child age (months)	0.00	0.06	0.00	0.955	0.955
	Internalizing symptoms (T1)	-0.06	0.08	-0.02	0.463	0.555
	Clinician-reported adverse birth events (T1)	-0.07	0.08	-0.07	0.396	0.555
	Clinician-reported adverse birth events × Internalizing symptoms (T1)	0.06	0.06	0.06	0.334	0.555
Positive parenting (T2)	Race (0 = White, 1 = Black)	-0.26	0.16	-0.14	0.097	0.291
	Relationship status	0.18	0.18	0.08	0.339	0.527
	Child age (months)	0.05	0.06	0.06	0.439	0.527
	Internalizing symptoms (T1)	-0.07	0.09	0.04	0.427	0.527
	Clinician-reported adverse birth events (T1)	0.00	0.09	0.00	0.992	0.992
	Clinician-reported adverse birth events × Internalizing symptoms (T1)	0.14	0.07	0.12	0.032*	0.191
Parenting stress (T3)	Race (0 = White, 1 = Black)	0.50	2.69	0.01	0.854	0.854
	Relationship status	3.07	3.50	0.07	0.381	0.793
	Child age (months)	-0.54	1.24	-0.03	0.661	0.793
	Internalizing symptoms (T1)	6.23	1.52	0.31	< 0.001*	< 0.001*
	Clinician-reported adverse birth events (T1)	1.07	1.54	0.05	0.487	0.793
	Clinician-reported adverse birth events × Internalizing symptoms (T1)	-0.69	1.21	-0.03	0.569	0.793

* indicates *p* < 0.05, ** indicates *p* < 0.01, *** indicates *p* < 0.001. *FDR p* = False discovery rate *p*-values. T1 = 12 weeks postpartum, T2 = 12 months postpartum, and T3 = 24 months postpartum. Mother infant bonding was assessed via the general impairment subscale of the Postpartum Bonding Questionnaire (PBQ), and parenting stress was measured via the Parenting Stress Index, fourth edition, short form (PSI-4-SF). Postpartum internalizing symptoms were assessed via a composite score of the Edinburgh Postnatal Depression Scale (EPDS) and Generalized Anxiety Disorder 7 (GAD-7). Mother-infant interactions and positive parenting were assessed via observational reports. Relationship status (1 = married/partnered, 0 = single/divorced/separated/widowed) and race (1 = Black, 0 = White) were dummy coded. The cumulative score of clinician-reported adverse childbirth events was derived based on the number of objective traumatic birth events recorded in the medical chart (i.e., caesarean-section, preterm-birth, significant blood loss, manual placental removal method, an Apgar score of less than 8, no labor analgesia, assisted vaginal births and/or episiotomy, and *≥*3rd degree lacerations/tearing)

### Aim 2b: Race-related differences in the relationship between clinician-reported adverse birth events on maternal caregiving

There were no differences in clinician-reported birth outcomes by race. Additionally, there were no significant interactions between childbirth trauma and race on maternal caregiving behaviors and attitudes. See Table 4.

## Discussion and conclusions

This investigation examined associations between childbirth trauma, measured by self-report and clinician-reported factors, and various caregiving behaviors and attitudes measured at 12 weeks, 12 months, and 24 months postpartum in a cohort of Black and White women who gave birth during the height of the COVID-19 pandemic. Our hypotheses were largely unsupported, as there was one positive association between postpartum individuals’ subjective birth experiences and caregiving behaviors measured at 12 months, but no other significant relationships were detected at 12 weeks or 24 months postpartum. Despite our prior work indirectly linking clinician-reported birth factors, such as cesarean section and blood loss to postpartum depression (Waller et al. 2022) , in this study, clinician-reported factors did not significantly predict maternal caregiving attitudes and behaviors. Lastly, although we found higher rates of subjective birth trauma in Black individuals, our results revealed no interactions with race, postpartum internalizing symptoms, and parenting outcomes.

The lack of significant associations and one positive association between subjective childbirth trauma and caregiving contributes to the mixed findings in the literature. Quantitative studies have mostly found that childbirth trauma is generally related to poor caregiving behaviors and attitudes, while more positive birth experiences are related to better caregiving outcomes (Bell et al. 2018a, b; Christie et al. 2019; Frankham et al. 2024). Relatedly, experiencing a traumatic childbirth compounded by postpartum internalizing symptoms, such as depression, can further hinder maternal caregiving abilities (Martini et al. 2022). Qualitative studies have also detailed how childbirth experiences can negatively shape mothers’ interactions with their infants (Beck and Watson 2019; Frankham et al. 2024; Molloy et al. 2021). Contrastingly, recent quantitative studies have failed to find significant, direct relationships between childbirth trauma and mother-infant bonding, attachment, and breastfeeding behaviors (Cruwys et al. 2024; Inzoli et al. 2021; Mollard and Kupzyk 2022). Two studies have even reported findings similar to ours, where traumatic childbirth experiences were associated with better mother-infant bonding, suggesting childbirth trauma may lead to an adaptive response that promotes caregiving behaviors and decreases negative feelings towards an infant.

Methodological differences may explain contrasting findings within the literature as well as the present study. First, childbirth trauma has been assessed in various ways, including the severity of one’s birth experience and postpartum birth-related or general PTSD symptoms. Differences in the measures used to describe birth experiences and PTSD symptoms may contribute to the mixed reports. This study created a 3-item measure that assessed the perception of trauma during childbirth but did not query individual symptoms, potentially artificially elevating the positive rate. Given the timing, during the early phase of the COVID pandemic, our assessment may have tapped into parents’ COVID-related fears that were not directly related to the childbirth experience. Despite these potential limitations, we have found that scores on this measure reliably predicted postpartum depression even after considering the history of depression and anxiety, indicating its validity (Waller et al. 2022). 

Similarly, methodological differences in the assessment of caregiving behaviors may contribute to the lack of consensus in the literature about how childbirth trauma relates to later caregiving behaviors. For example, differences in the timing of assessment of childbirth trauma and caregiving behaviors may contribute to equivocal findings. Quantitative reports have measured maternal caregiving behaviors from immediately after birth up to 14 months postpartum (Döblin et al. 2023). Some studies have found that caregiving behaviors measured during the later stages of child development were not significantly associated with childbirth trauma (Bell et al. 2018a, b; Döblin et al. 2023). It is possible that birth experiences may influence early maternal behaviors but not those measured at later stages as the traumatic experience becomes less salient with time or treatment. Notably, our first assessment of caregiving and birth trauma was at 12 weeks postpartum, which is within the same time frame that previous studies have failed to find an effect. Assessment at 12 weeks, as well as 12 and 24 months postpartum, might already have been too late to see the impact of childbirth trauma on caregiving. Nonetheless, our data did show a longitudinal effect with reports at 12 weeks and 12 months postpartum. One possible explanation is that the parenting assessment type used at 12 months was observational, while previous studies have typically used self-report measures. Perhaps there is a difference in how mothers rate their parenting behavior vs. observational measures.

Study strengths include our examination of a diverse cohort of Black and White women who gave birth during the beginning of the COVID-19 pandemic, one of the most uncertain times in recent history. This novel investigation adds to the literature by including an underrepresented group (Black women), building on research which suggests they are at highest risk of experiencing traumatic births (Bell et al. 2018a, b; Cook et al. 2017; Markin and Coleman 2023). We did not find a significant interaction between race and birth trauma, suggesting that race may not exacerbate the association between birth trauma and caregiving behaviors. However, due to sample size, it is possible we were not able to detect significant differences. Nonetheless, the present study’s novel inclusion of Black participant’s outcomes compared to White participants helps explain how traumatic birth experiences impact women who are most vulnerable to childbirth trauma and generalize findings to more groups of people. More research is needed to examine the relationship between traumatic birth experiences and subsequent caregiving and parenting behaviors in diverse cohorts.

Another strength includes utilizing EHR data to examine objective traumatic birth experiences, such as having a c-section or experiencing severe blood loss. Previous work has highlighted that these adverse birth events can influence perceived birth experience (Horsch et al. 2024; Mergler et al. 2025; Waller et al. 2022). Other strengths include our longitudinal design across 12 weeks to 24 months postpartum as well as the novel use of observed measures of caregiving behaviors. Observed mother-child interactions are considered the gold standard in assessment, however, are infrequently used due to time, costs, and other burden associated with observation and coding. These assessments often yield different results than self-report measures, which are more commonly used (Zahidi et al. 2019).

The present study is not without limitations, and results must be interpreted with caution. First, this investigation is a retrospective analysis of a longitudinal study. Several of the dependent variables examined were not assessed at multiple timepoints. Additionally, the number of participants varies at the different timepoints, and our sample size may have been underpowered to detect significant relationships. Second, this sample is a normative convenience sample, and participants generally reported less traumatic birth experiences. Readers should also be aware that our 3-item self-report of childbirth-related trauma was assessed at 12 weeks postpartum, and we may have missed self-reported trauma within the acute postpartum phase. Similarly, our assessment, designed for brevity, may have missed specific symptoms that would have resulted in a higher prevalence or reported severity of self-reported trauma. Third, our measure of clinician-reported adverse childbirth events is not exhaustive of all possible childbirth complications and is limited by available data. Lastly, our data was collected during COVID-19 pandemic. Because women were pregnant and postpartum during a national crisis, our findings may not be comparable to previous studies conducted prior to March 2020. The pandemic could have influenced how traumatized (or not) women felt towards their pregnancy. Pregnant individuals may have normalized a traumatic birth due to news, stories from friends and families, and research. Additionally, quarantine and isolation practices induced by the COVID-10 pandemic may have allowed mothers more opportunity to spend time with their children, influencing their quality of mother-infant bonding and interactions.

## Conclusion

Contrary to prior research, childbirth trauma was not significantly associated with poorer maternal caregiving behaviors and attitudes in our sample. The present study found the opposite relationship: more traumatic childbirths were significantly associated with maternal positive parenting. More research is warranted to untangle these conflicting findings and understand the complex interplay between delivery experiences and later caregiving behaviors. This study extends the literature by including Black women as they have been previously underrepresented in this body of research. Future research must continue to include minoritized women in these types of studies to ensure diverse experiences of childbirth trauma are explored.

## Supplementary Information

Below is the link to the electronic supplementary material.

Supplementary Material 1

## Data Availability

Data may be obtained with permission of the authors and after reaching out to the corresponding author.

## Declarations

### Registered protocol

There is no registered protocol for this study.
