# Supplementary material for: Traumatic birth experiences and maternal caregiving behaviors and attitudes in black and white women
Source: Arch Womens Ment Health. 2026 Mar 19;29(2):51. doi: 10.1007/s00737-026-01682-6 (PMC12999831; doi:10.1007/s00737-026-01682-6)
Supplement: Supplementary file 1 — Supplementary Material 1 [file 737_2026_1682_MOESM1_ESM.docx]

**Supplemental Materials**

**Traumatic birth experiences and maternal caregiving behaviors and attitudes in Black and White women**

| **Table S1**  *Description of study variables* | | | | |
| --- | --- | --- | --- | --- |
| **Variable** | Timepoint | Measure | Description | Valid data (*n*) |
| **Race** | Pregnancy, T1, or T2 | Demographic survey/EHR | At T1 and T2, mothers reported their racial identity. A total of 44 participants did not report their race in the T1 and/or T2 survey. For these participants, race was identified from EHR.  One caveat with using EHR for racial identification is that there is not always a standardized method of obtaining this information (Kim et al., 2022). Health professionals may assume someone’s race, which may not reflect one’s self-reported identity. | All participants |
| **Self-reported birth trauma** | T1 | 3-item survey (created by study authors) | Self-reported birth trauma was reported from three questions on a scale of 1 (*Not at all*) to 4 (*Extremely/a lot*): (1) “During your delivery or immediately after delivery, how much did you fear for your own life?”, (2) “During your delivery or immediately after delivery, how much did you fear for your baby's life?”, and (3) “How traumatic did you find your birth experience?”. These questions developed by a team of psychologists and psychiatrist and were based on Criterion A from the DSM-5 diagnostic criteria for posttraumatic stress disorder (Hyland et al., 2018). The sum of the three items calculates birth trauma. Higher scores indicate more traumatic birth experiences. Internal validity was good (Cronbach’s alpha = .76), the measure was significantly correlated to several clinical-reported adverse birth events, such as having a preterm birth or not or caesarean-section (See Table S1). See [BLINDED FOR REVIEW] for additional details of self-reported birth trauma. | All participants |
| **Caregiving behaviors and attitudes** | | | | |
| **Self-reported mother-infant bonding** | T1 | “General impairment” subscale from Postpartum Bonding Questionnaire (PBQ; Brockington et al., 2006) | The PBQ “general impairment” subscale consists of 12 questions that can be scored from 1 (*Always*) to 6 (*Never*) and was assessed at T1. Participants were asked questions about feelings towards their baby (e.g., “I feel close to my baby”) and attitudes about being a mother (e.g., “I feel trapped as a mother”). Internal validity was good (Cronbach’s alpha = .80). | 253 |
| **Observed mother-infant interactions** | T2 | Observational ratings | Coders assessed mother-infant interactions from video recordings of birthing individuals and their children engaging in various tasks (i.e., storybook, teaching, free play). Interactions were assessed at T2, and this construct measures the level of synchrony, comfort, and mutual pleasure between parent and child while participating in the three tasks. Coders rated interactions on a scale 0 (*Very low*) to 7 (*Very high*) for all three tasks. A confirmatory factor analysis derived a score of observed mother-infant interactions. Low scores indicate that a dyad operates as two separate entities, while high scores indicate a high degree of synchrony and comfort. | 163 |
| **Observed positive parenting** | T2 | Observational ratings | Positive parenting was measured at T2 and T3 via the video recordings and was assessed using ratings from the three tasks (i.e., storybook, teaching, and free play tasks). The three facets of positive parenting include maternal sensitivity (i.e., the parent ability to appropriately respond to child’s cues during times of distress and non-distress), positive regard (i.e., the parent’s praise and love/admiration for their child), and stimulation of cognitive development (i.e., the parent’s effortful teaching to enhance perceptual, cognitive, and linguistic development). Coders rated each facet on a scale of 0 (*Very low*) to 7 (*Very high*) for all three tasks. A confirmatory factor analysis derived a score of observed positive parenting. Higher scores indicate high levels of positive parenting. See [BLINDED FOR REVIEW] for additional psychometric details. | 172 |
| **Self-reported parenting stress** | T3 | Parenting Stress Index, fourth edition, short form (PSI-4-SF; Barroso et al., 2016) | The PSI-4-SF measures the amount of stress present in parent-child relationships. This short form uses 36 items from the original version of the PSI (Barroso et al., 2016). Items are rated on a scale of 1 (*Strongly disagree*) to 5 (*Strongly agree*). Internal validity was excellent (Cronbach’s alpha = .92). | 211 |
| **Baseline sociodemographic and delivery information** | | | | |
| **Internalizing symptoms** | T1 | Edinburgh Postnatal Depression (EPDS, Cox, 2017; Cox et al., 1987), Generalized Anxiety Disorder 7-item Scale (GAD-7; Spitzer et al., 2006) | Depression was measured via the EPDS, which is a 10-item screener that assesses depression symptoms in postpartum parents. Internal validity was good (Cronbach’s alpha = .87). Anxiety was measured via the GAD-7, which measures the frequency of one’s anxiety symptoms over the past two weeks. Internal validity was excellent (Cronbach’s alpha = .91). The EPDS and GAD-7 were standardized, and the average of the two were taken to create a composite internalizing symptoms score. | All participants |
| **Relationship status** | T1 | Demographics survey | Participants indicated their current relationship status as partnered/married, divorced/separated, widowed, single, or other. | 253 |
| **Delivery method** | Birth | EHR | Participants were categorized as either having a vaginal or cesarean-section delivery. | All participants |
| **Preterm birth** | Birth | EHR | Participants were characterized as a having a preterm birth if they delivered at 36 gestational weeks or less. | All participants |
| **Placenta removal method** | Birth | EHR | Placental removal method was categorized as having a manual removal or spontaneous/expressed removal. | All participants |
| **Blood loss (mL)** | Birth | EHR | Blood loss refers to the estimated amount of blood a birthing individual lost during delivery and is measured in milliliters. | 169 |
| **Labor analgesia** | Birth | EHR | Participants were categorized as receiving labor analgesia (i.e., general, spinal, epidural) or not. | 252 |
| **Assisted vaginal births and/or episiotomy** | Birth | EHR | Participants were characterized as having an assisted vaginal birth (e.g., forceps, vacuum) and/or episiotomy or not. | 169 |
| **Lacerations/ Tearing** | Birth | EHR | Lacerations were categorized by the degree of tearing: no recorded lacerations, 1^st^ degree, 2^nd^ degree, 3^rd^ degree, and 4^th^ degree. | 196 |
| **Cumulative score of clinician-reported adverse childbirth events** | Birth | EHR | A cumulative score of clinician-reported adverse childbirth events was derived based on the number of objective traumatic birth events recorded in the medical chart (i.e., caesarean-section, preterm-birth, significant blood loss, placental removal method, a 5-min Apgar score of less than 8, assisted vaginal birth and/or episiotomy, lacerations/tearing, and no labor analgesia). A higher score indicates that a participant experienced more clinician-reported adverse childbirth events. | All participants |

| **Table S2**  *Correlation matrix of all study variables* | | | | | | | | | | | | | | | | | | | | | |
| --- | --- | --- | --- | --- | --- | --- | --- | --- | --- | --- | --- | --- | --- | --- | --- | --- | --- | --- | --- | --- | --- |
| Variable | 1 | 2 | 3 | 4 | 5 | 6 | 7 | 8 | 9 | 10 | 11 | 12 | 13 | 14 | 15 | 16 | 17 | 18 | 19 | 20 | 21 |
| 1. Self-reported birth trauma (T1) | 1.00 |  |  |  |  |  |  |  |  |  |  |  |  |  |  |  |  |  |  |  |  |
| 1. Mother-infant bonding (T1) | 0.05 | 1.00 |  |  |  |  |  |  |  |  |  |  |  |  |  |  |  |  |  |  |  |
| 1. Mother-infant interactions (T2) | 0.15* | -0.13 | 1.00 |  |  |  |  |  |  |  |  |  |  |  |  |  |  |  |  |  |  |
| 1. Positive parenting (T2) | 0.11 | -0.05 | 0.51*** | 1.00 |  |  |  |  |  |  |  |  |  |  |  |  |  |  |  |  |  |
| 1. Parenting stress (T3) | -0.02 | 0.44*** | 0.01 | 0.01 | 1.00 |  |  |  |  |  |  |  |  |  |  |  |  |  |  |  |  |
| 1. Internalizing symptoms (T1) | 0.15* | 0.33*** | -0.01 | 0.07 | 0.33*** | 1.00 |  |  |  |  |  |  |  |  |  |  |  |  |  |  |  |
| 1. Income (T1) | -0.21*** | 0.18** | -0.19* | 0.10 | -0.04 | 0.01 | 1.00 |  |  |  |  |  |  |  |  |  |  |  |  |  |  |
| 1. Education (T1) | -0.11 | 0.13* | 0.04 | 0.22** | -0.05 | 0.02 | 0.76*** | 1.00 |  |  |  |  |  |  |  |  |  |  |  |  |  |
| 1. Relationship status (T1) | 0.17** | 0.08 | 0.01 | -0.16* | 0.04 | 0.06 | -0.12 | -0.11 | 1.00 |  |  |  |  |  |  |  |  |  |  |  |  |
| 1. Maternal age at birth | -0.12 | 0.09 | -0.09 | -0.02 | 0.02 | -0.03 | 0.43*** | 0.43*** | -0.13* | 1.00 |  |  |  |  |  |  |  |  |  |  |  |
| 1. Child age (T1) | 0.13* | -0.09 | -0.09 | 0.01 | -0.07 | 0.13* | -0.11 | -0.10 | -0.03 | -0.09 | 1.00 |  |  |  |  |  |  |  |  |  |  |
| 1. Child age (T2) | 0.05 | -0.06 | 0.00 | 0.01 | 0.08 | 0.01 | -0.14 | -0.16* | -0.03 | -0.02 | 0.20** | 1.00 |  |  |  |  |  |  |  |  |  |
| 1. Child age (T3) | 0.09 | -0.10 | -0.03 | -0.05 | -0.07 | -0.10 | -0.10 | -0.14* | 0.12 | 0.09 | 0.26*** | 0.18 | 1.00 |  |  |  |  |  |  |  |  |
| 1. Preterm birth | 0.11 | 0.06 | 0.10 | 0.09 | 0.07 | -0.01 | 0.03 | 0.05 | -0.03 | 0.12* | 0.03 | -0.06 | 0.03 | 1.00 |  |  |  |  |  |  |  |
| 1. Delivery method | 0.26*** | 0.08 | -0.08 | -0.09 | 0.03 | 0.00 | 0.05 | 0.07 | 0.04 | 0.25*** | -0.06 | -0.01 | 0.03 | 0.10 | 1.00 |  |  |  |  |  |  |
| 1. Placental removal method | 0.03 | 0.01 | 0.00 | -0.02 | -0.09 | 0.01 | 0.02 | 0.01 | -0.02 | 0.01 | 0.03 | 0.03 | 0.05 | 0.01 | -0.15* | 1.00 |  |  |  |  |  |
| 1. 5 min Apgar score at birth | -0.20* | -0.03 | -0.05 | -0.03 | -0.07 | 0.01 | 0.19** | 0.08 | -0.14* | 0.04 | -0.06 | -0.02 | -0.03 | -0.13* | -0.22*** | 0.05 | 1.00 |  |  |  |  |
| 1. Blood loss at birth | 0.08 | -0.16* | -0.09 | 0.09 | -0.20* | -0.13 | -0.01 | 0.08 | -0.04 | -0.12 | 0.02 | 0.06 | 0.00 | -0.05 | 0.17* | 0.20** | -0.17* | 1.00 |  |  |  |
| 1. Labor analgesia | -0.07 | 0.07 | -0.03 | -0.12 | 0.20** | 0.06 | -0.05 | -0.09 | -0.03 | -0.02 | -0.01 | 0.01 | 0.04 | 0.08 | -0.19* | -0.07 | 0.05 | -0.19* | 1.00 |  |  |
| 1. Assisted vaginal births and/or episiotomy | 0.16* | 0.04 | -0.10 | 0.14 | 0.20* | 0.06 | -0.07 | -0.01 | -0.02 | -0.10 | -0.10 | 0.11 | -0.07 | -0.08 | -0.13 | 0.05 | -0.08 | 0.21* | -0.09 | 1.00 |  |
| 1. Lacerations/tearing | 0.08 | 0.07 | 0.04 | 0.16 | -0.04 | 0.14* | 0.08 | 0.13 | -0.01 | -0.01 | 0.01 | 0.06 | 0.02 | -0.05 | -0.07 | 0.18* | 0.04 | 0.37*** | -0.07 | 0.23** | 1.00 |
| 1. Clinician-reported adverse childbirth events | 0.28*** | 0.06 | -0.05 | 0.02 | 0.08 | 0.05 | -0.04 | 0.03 | 0.03 | 0.08 | -0.01 | 0.05 | 0.05 | 0.39*** | 0.44*** | 0.28*** | -0.44*** | 0.51*** | 0.18** | 0.39*** | 0.40*** |
| ***Note.*** * indicates *p* < .05, ** indicates *p* < .01, *** indicates *p* < .001. T1 = 12 weeks postpartum, T2 = 12 months postpartum, and T3 = 24 months postpartum. Mother infant bonding was assessed via the general impairment subscale of the Postpartum Bonding Questionnaire (PBQ), and parenting stress was measured via the Parenting Stress Index, fourth edition, short form (PSI-4-SF). Birth trauma was assessed via questionnaire created by the study’s authors. Baseline internalizing symptoms were assessed via a composite score of the Edinburgh Postnatal Depression Scale (EPDS) and Generalized Anxiety Disorder 7 (GAD-7). Mother-infant interactions and positive parenting were assessed via observational reports. Relationship status (1 = married/partnered, 0 = single/divorced/separated/widowed) and race (1 = Black, 0 = White) were dummy coded. The cumulative score of clinician-reported adverse childbirth events was derived based on the number of objective traumatic birth events recorded in the medical chart (i.e., caesarean-section, preterm-birth, significant blood loss, manual placental removal method, an Apgar score of less than 8, no labor analgesia, assisted vaginal births and/or episiotomy, and >3^rd^ degree lacerations/tearing). | | | | | | | | | | | | | | | | | | | | | |

**References**

Barroso, N. E., Hungerford, G. M., Garcia, D., Graziano, P. A., & Bagner, D. M. (2016). Psychometric properties of the Parenting Stress Index-Short Form (PSI-SF) in a high-risk sample of mothers and their infants. *Psychological Assessment*, *28*(10), 1331–1335. https://doi.org/10.1037/pas0000257

Brockington, I. F., Fraser, C., & Wilson, D. (2006). The Postpartum Bonding Questionnaire: A validation. *Archives of Women’s Mental Health*, *9*(5), 233–242. https://doi.org/10.1007/s00737-006-0132-1

Cox, J. (2017). Use and misuse of the Edinburgh Postnatal Depression Scale (EPDS): A ten point ‘survival analysis.’ *Archives of Women’s Mental Health*, *20*, 789–790.

Cox, J. L., Holden, J. M., & Sagovsky, R. (1987). Detection of postnatal depression: Development of the 10-item Edinburgh Postnatal Depression Scale. *The British Journal of Psychiatry*, *150*(6), 782–786.

Hyland, P., Shevlin, M., Fyvie, C., & Karatzias, T. (2018). Posttraumatic Stress Disorder and Complex Posttraumatic Stress Disorder in *DSM‐5* and *ICD‐11*: Clinical and Behavioral Correlates. *Journal of Traumatic Stress*, *31*(2), 174–180. https://doi.org/10.1002/jts.22272

Kim, Y., Pirritano, M., & Parrish, K. M. (2022). Determinants of racial and ethnic disparities in utilization of hospital-based care for asthma among Medi-Cal children in Los Angeles. *The Journal of Asthma : Official Journal of the Association for the Care of Asthma*, *59*(8), 1521–1530. https://doi.org/10.1080/02770903.2021.1955131

Spitzer, R. L., Kroenke, K., Williams, J. B., & Löwe, B. (2006). A brief measure for assessing generalized anxiety disorder: The GAD-7. *Archives of Internal Medicine*, *166*(10), 1092–1097.

Waller R, Kornfield SL, White LK, Chaiyachati BH, Barzilay R, Njoroge W, Parish-Morris J, Duncan A, Himes MM, Rodriguez Y, Seidlitz J, Riis V, Burris HH, Gur RE, & Elovitz MA. (2022). Clinician-reported childbirth outcomes, patient-reported childbirth trauma, and risk for postpartum depression. *Arch Womens Ment Health*, *25*(5), 985–993. https://doi.org/10.1007/s00737-022-01263-3
